# Case Report: Intraoperative endoscopic treatment for the “pseudo” lumen formation of jejuno-esophageal anastomosis in laparoscopic total gastrectomy

**DOI:** 10.3389/fonc.2025.1513844

**Published:** 2025-04-28

**Authors:** Zhixiong Chen, Xiaojie Gao, Jingkun Shang, Jieli Huang, Shouru Zhang

**Affiliations:** Department of Gastrointestinal Tumor Center, Chongqing University Cancer Hospital, Chongqing, China

**Keywords:** laparoscopy, adenocarcinoma of esophagogastric junction (AEG), anastomosis, complications, self-expanding covered metal stent

## Abstract

Recently, the incidence of adenocarcinoma of esophagogastric junction (AEG) is increasing in China. Laparoscopic total gastrectomy plus D2 lymph node dissection is an important treatment method for AEG of Siewert type II. In order to achieve the esophagojejunostomy, surgical techniques, no matter in open surgery or in laparoscopic one, are highly demanding. This report describes a case of surgical complication during laparoscopic total gastrectomy, in which a pseudoaneurysm developed at the gastroesophageal junction during anastomosis. The condition was successfully managed with endoscopic mucosal incision and placement of a self-expanding covered metal stent (SEMs) as a salvage intervention. The purpose of this report is to share an clinical treatment experience of similar cases, and at the same time to warn surgeon to perform esophagojejunostomy carefully.

## Introduction

There is an upward trend in the incidence of adenocarcinoma of the esophagogastric junction (AEG) (1, 2). Surgery has become an important treatment for AEG. Laparoscopic radical surgery has rapidly advanced with technological and equipment improvements, providing benefits such as enhanced field of view, reduced postoperative pain, smaller incisions, and quicker recovery. However, performing this technique is relatively difficult, especially in the reconstruction of esophagojejunal anastomosis after proximal gastrectomy or total gastrectomy. There are several types of AEG based on the relationship between tumor center and dentate line. Siewert typing is more commonly used. Siewert III surgery is certainly not difficult, and even total laparoscopic gastrectomy and digestive tract reconstruction are superior to open surgery (3). However, for some Siewert II tumors with a higher location, ensuring a safe incision margin and overcoming anatomical challenges such as obstruction by the bilateral diaphragmatic crura and a narrow esophageal hiatus significantly increase the difficulty of exposure and manipulation during esophagojejunal anastomosis, leading to a higher risk of intraoperative adverse clinical events, including “pseudo” lumen formation of anastomosis, excessive anastomotic tension, and imprecise suturing, which may result in postoperative anastomotic leakage. If not treated or not detected in a timely manner, these complications can lead to serious consequences. Given the advanced endoscopic operation platform at our center, along with skilled surgeons and extensive experience in treating esophageal fistula and AEG after neoadjuvant chemoradiotherapy using self-expanding covered metal stents (SEMs), we present a successful case of avoiding mid-operation conversion to open chest/thoracoscopic assisted surgery. This was achieved by performing endoscopic resection of the esophageal “pseudolumen” and placing SEMs in an AEG patient undergoing laparoscopic side-to-side anastomosis of the jejunum to the esophagus using a side-viewing endoscope during the procedure. The purpose of this report is to share clinical treatment experience in similar cases and remind specialist surgeons to ensure accurate entry of the clamp arm into the mucosa of the esophagus and/or jejunum before closing the common opening of the endoscopic jejunum-esophagus overlap anastomosis using a side-viewing endoscope.

## Case presentation

**Figure 1:**
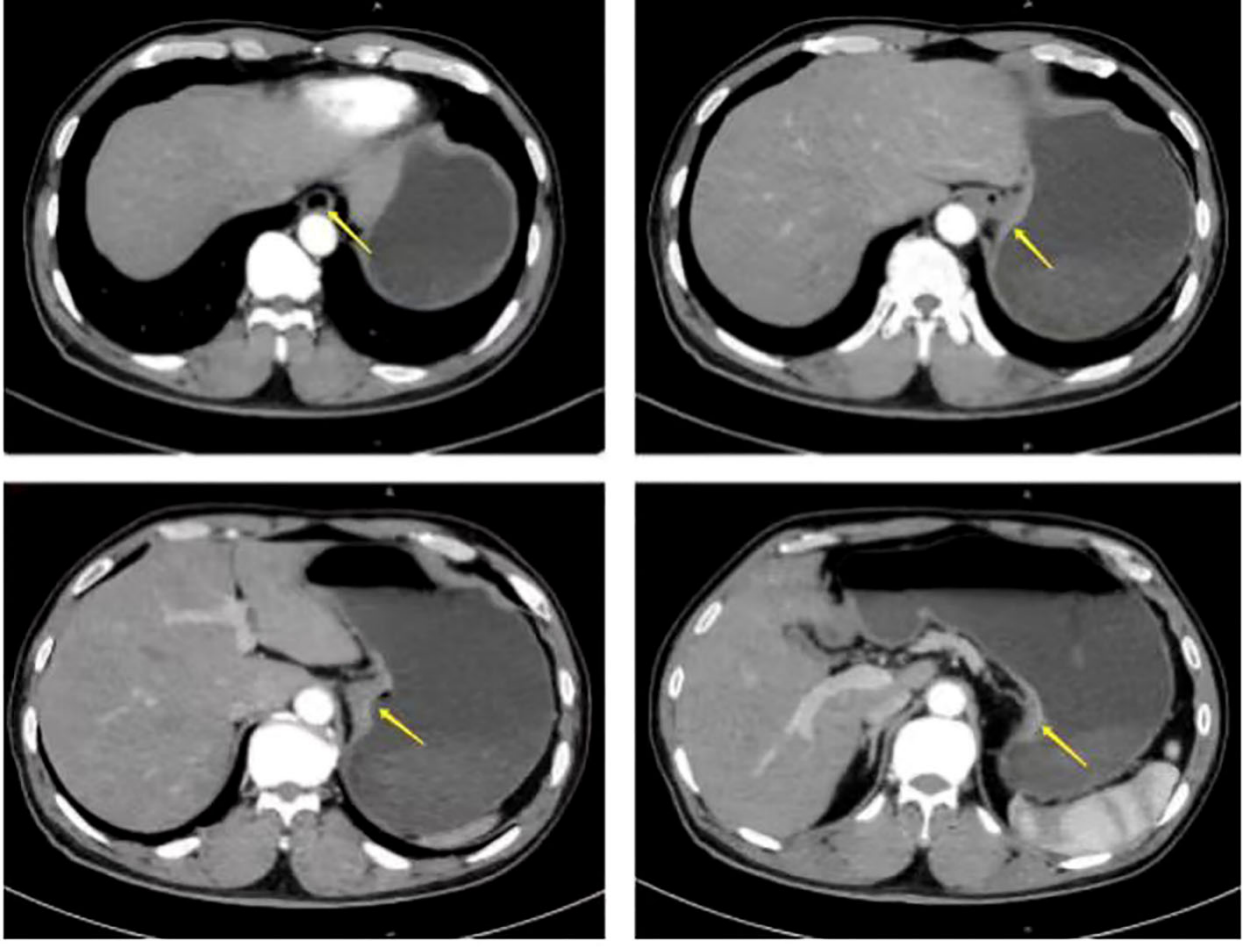
Preoperative computed tomography (CT) scan showed the thickening and enhancement suggestive of malignancy near the gastroesophageal junction but without evidence of regional lymph node involvement or distant spread (arrow).

**Figure 2:**
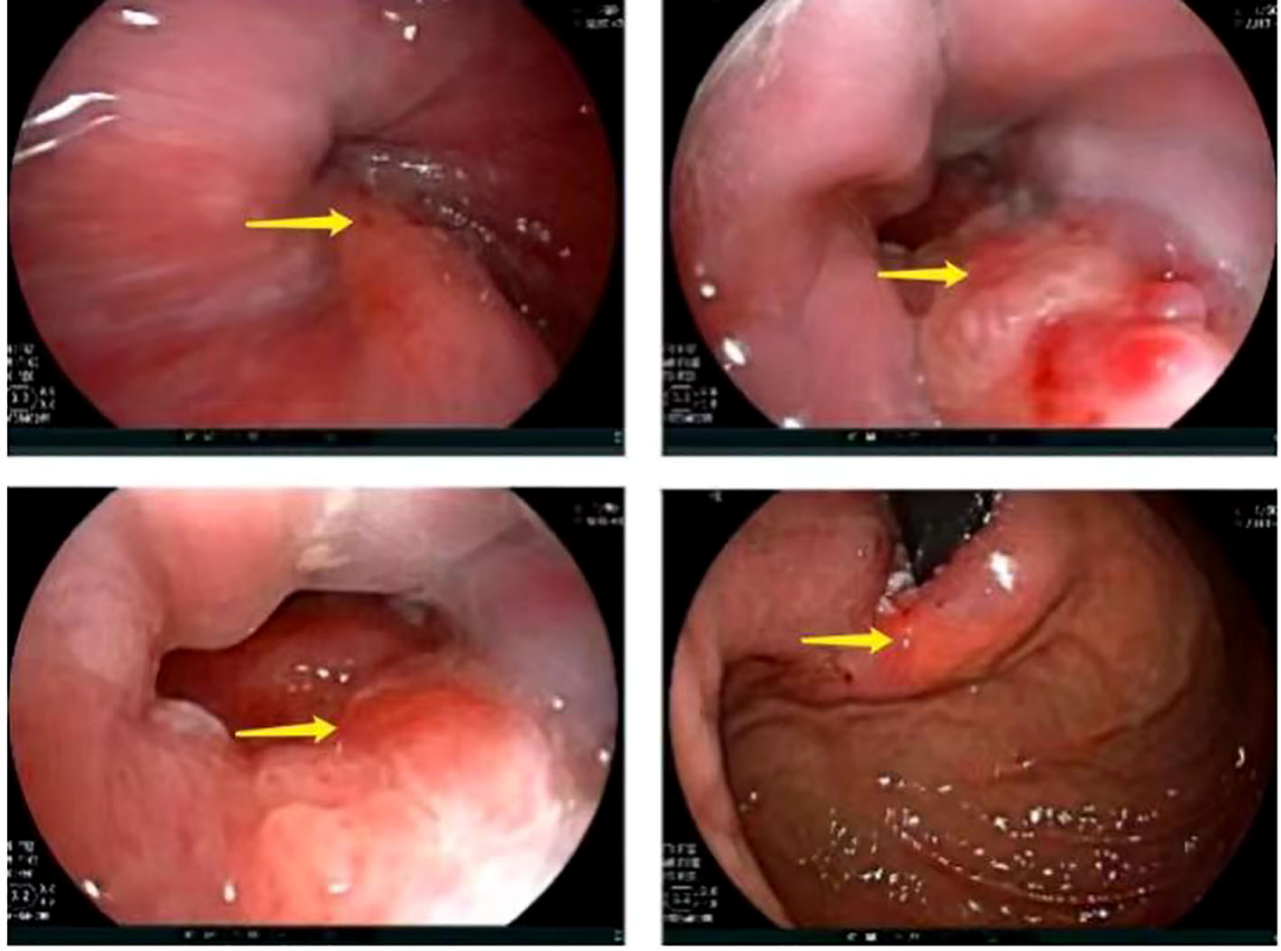
Preoperative gastroscopic examination revealed a tumor located at the gastroesophageal junction, approximately 1.5 cm above the Siewert II type esophagogastric junction (arrow).

A 67-year-old male presented with sternal discomfort following meals for two weeks. Initial evaluation at a local hospital included endoscopy and subsequent biopsy. The former report revealed abnormal mucosal changes in the distal esophagus about 39cm from the incisor and malignancy cannot be excluded. In addition, the endoscope was able to enter the stomach smoothly and no abnormalities were found in the rest of the stomach and duodenum. The subsequent biopsy indicated high-grade intramucosal neoplasia. For him, there was no evidence of metastasis or familial predisposition to cancer. Upon referral to our facility for further management, physical examination yielded no significant findings while computed tomography (CT) scan (**Figure 1**) demonstrated thickening and enhancement suggestive of malignancy near the gastroesophageal junction but without evidence of regional lymph node involvement or distant spread. The endoscopic examination revealed a tumor located at the gastroesophageal junction (**Figure 2**), approximately 1.5 cm above the Siewert II type esophagogastric junction, and the biopsy indicated adenocarcinoma with Her2 (-). The initial diagnosis upon admission was Siewert II type esophagogastric junction adenocarcinoma, and clinic stage is cT3NxM0, II or higher.

**Figure 3:**
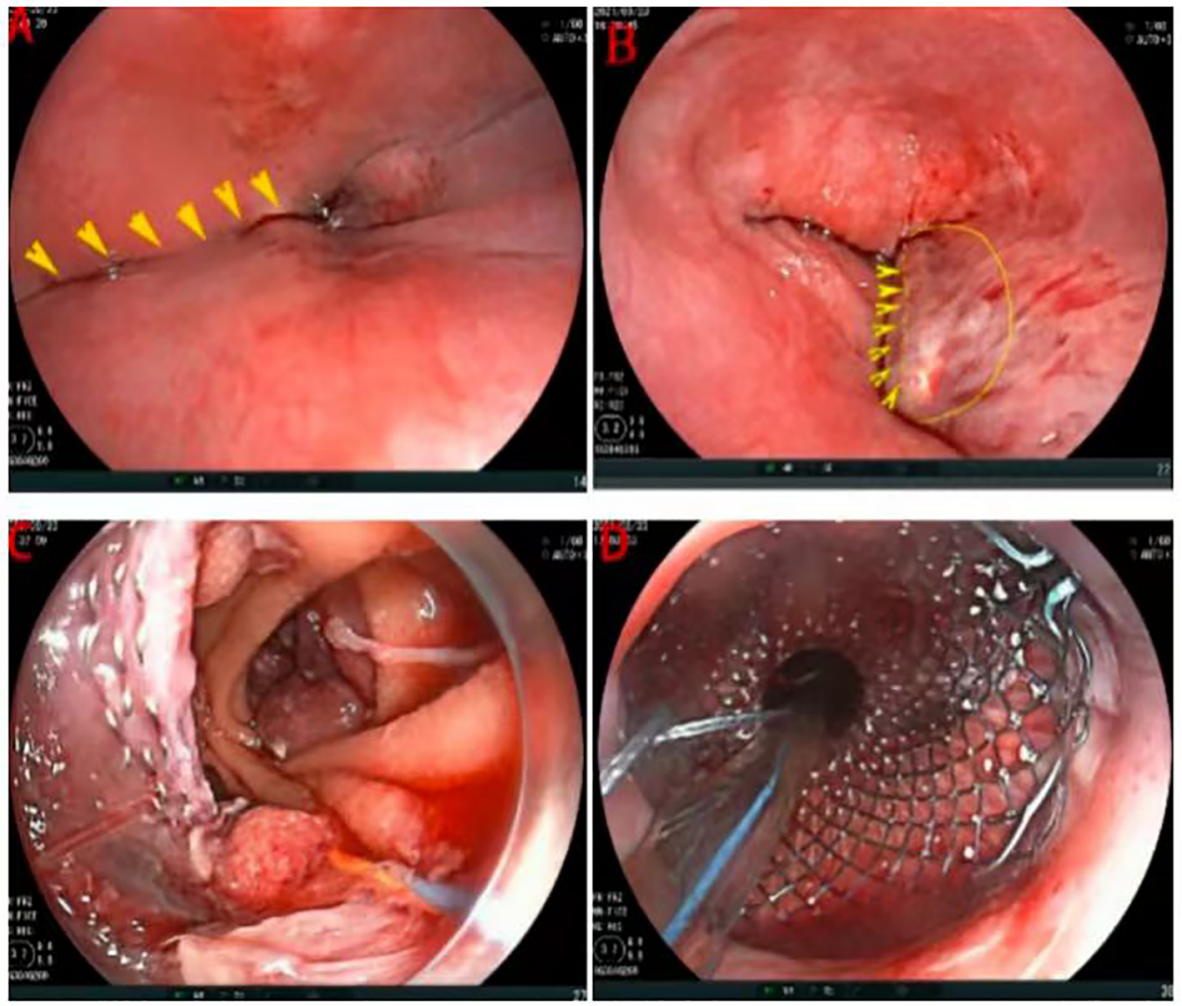
Intraoperative gastroscopic examination and operation. **(A)** The stapled segment of the esophageal stump (arrow). **(B)** Cutting the mucosa of the stoma canal (arrow). **(C)** After cutting the mucosa of the stoma canal, enabling easy placement of the scope into the small intestinal lumen. **(D)** Deploying SEMs and placing a jejunal feeding tube.

The patient declined neoadjuvant therapy and chose to undergo laparoscopic total gastrectomy with D2 lymphadenectomy (digestive tract reconstruction method: endoscopic esophagus-jejunal overlap anastomosis) under general anesthesia on September 23, 2021 following preoperative preparation and anesthetic assessment. During the procedure, the Overlap anastomosis was performed using a linear cutting and closure device under laparoscopy. However, after completing the closure of the common orifice, it was discovered that the feeding tube could not be inserted into the small intestine. The thoracic surgeon, endoscopist, and anesthesiologist held a consultation immediately during the operation: gastroscopy examination revealed that the anastomosis formed a pseudo-lumen between the jejunum and esophagus (**Figures 3A, B**). Subsequently, the operation went on. During laparoscopic dissection, the common staple line was incised by using clod knife to facilitate laparoscopic guidance. An endoscope (FJUIFILM, Processor VP-3500HD; EG-580RD) equipped with a disposable mucosal incision device (Olympus, KD-612L) was utilized to cut the mucosa of the stoma canal (**Figure 3B**), enabling easy placement of the scope into the small intestinal lumen (**Figure 3C**). Then, a zebra guidewire (Boston Scientific Corporation, M00556581/M00; 0.035 inch * 450 cm) was passed through the endoscope clamping port and advanced to the anastomotic site distal to the caecum. Subsequently, a SEMs pusher [Nanjing Microcreate, JSMA-2-18080-080; φ18mm, 60mm long] was introduced through the mouth using the guidewire and successfully deployed SEMs under endoscopic visualization at the anastomotic site. Finally, a jejunal feeding tube was placed for postoperative enteral nutrition (**Figure 3D**).

**Figure 4:**
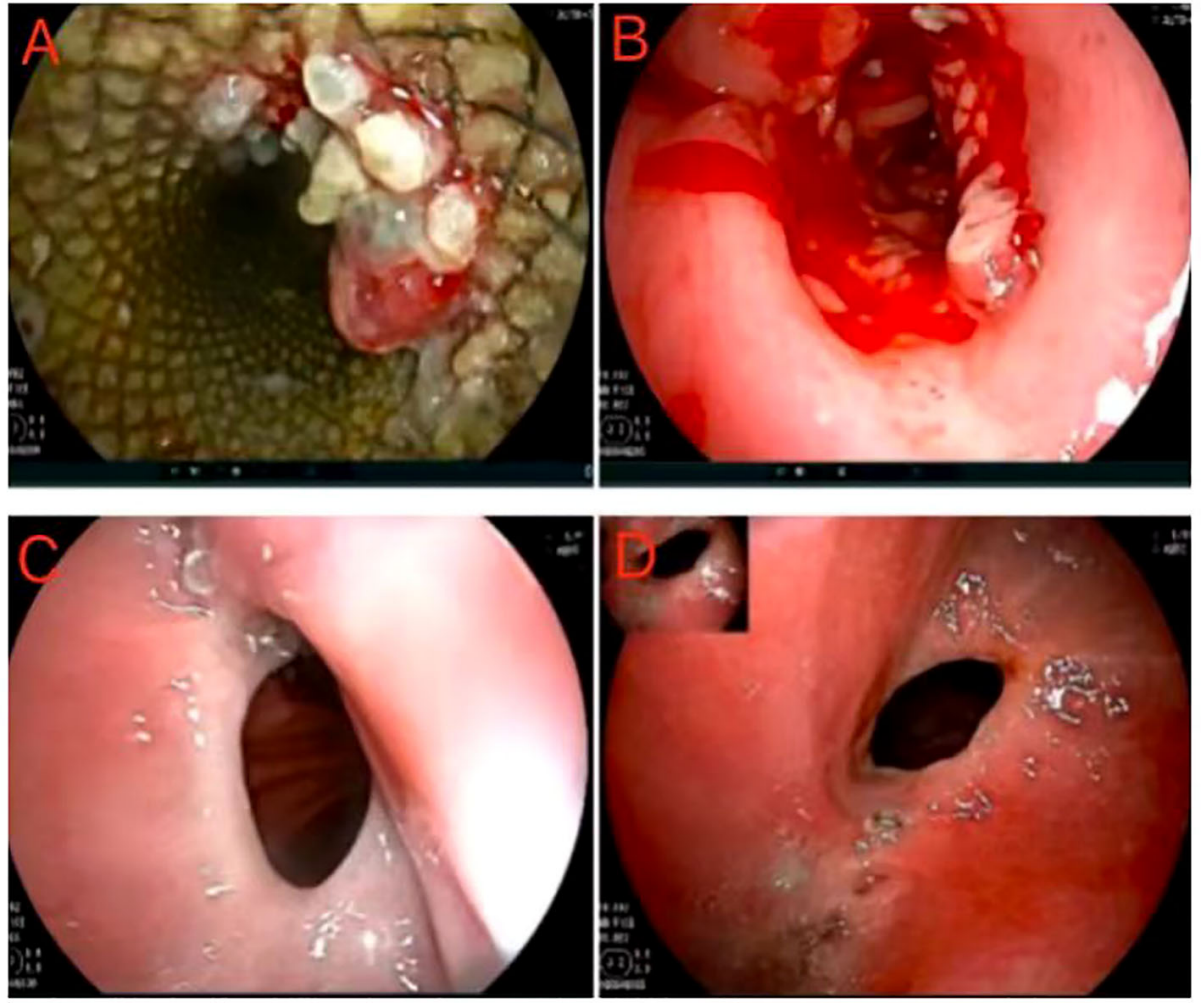
Postoperative gastroscopic examination and operation. **(A, B)** Postoperative first follow-up and stent removal. **(C)** Postoperative second follow-up, and endoscopy revealed mild stenosis atthe anastomosis. **(D)** Postoperative third follow-up, and the anastomotic stoma showed no significant changes.

After undergoing surgical intervention, the patient received short-term parenteral nutrition support and subsequently transitioned to enteral nutrition. The pathological examination revealed the following findings: a cardiac ulcer mixed adenocarcinoma involving both the entire stomach and tumor, with low-adherent adenocarcinoma constituting approximately 90% and tubular adenocarcinoma constituting about 10%. The patient underwent SOX chemotherapy on October 12 and November 5, 2021 and an endoscopy was performed on November 9 for stent removal (**Figures 4A, B**). Subsequent hospital visits occurred on November 28th, 2011, December 25th, 2011 and January 18th, 2022 for additional SOX chemotherapy sessions. On January 21st, 2022, endoscopy revealed mild stenosis at the anastomosis (**Figure 4C**), but no obvious difficulty swallowing semi-liquid or slowly eating solid foods was reported by the patient. After that, the patient declined further chemotherapy. On June 16th,2022, the patient was readmitted for another endoscopy which showed no significant changes (**Figure 4D**). In addition, no abnormalities were found in tumor markers or chest/abdominal enhanced CT scans. As of manuscript submission for follow-up, the patient has reported no abnormalities.

## Discussion

The optimal surgical approach for Seiwert II type of adenocarcinoma of the esophagogastric junction (AEG) remains a subject of debate, particularly with regard to surgical technique and method (4). Clinical trials have demonstrated that laparoscopic total gastrectomy is non-inferior to open surgery in terms of both efficacy and safety, establishing it as a crucial surgical option for AEG based on findings from the JCOG9502 trial and the lymph node metastasis pattern (4, 5). The 6^th^ edition of the Japanese *Guidelines for Gastric Cancer Treatment* recommends the abdominal transhiatal approach (TH) as the primary approach when esophageal invasion length is less than 3cm. Among various techniques for gastrointestinal tract reconstruction, laparoscopic esophagojejunal overlap anastomosis is currently widely discussed but requires advanced technical skills (6). Traditional overlap anastomosis has drawbacks such as excessive traction on the esophageal stump by instruments, leading to potential complications including injury. Except that, the complexity of esophageal retraction, in conjunction with the limited operative space, necessitates a three-dimensional approach to the procedure: esophageal/jejunal traction, exposure of the operative field, and simultaneous adjustment of the linear cutting clamp’s peg rotation/advance/retreat angle. This can potentially lead to various complications. In detail, a punch biopsy can inadvertently enter a “pseudo” lumen between the mucosa and muscle layer of the esophagus, and then puncture the side of a perforated segment of the small intestine or the wall of the esophagus, or cause mechanical injury to the esophagus through tearing or fixation with sutures. Others include the insufficiency of cutting and closing range(easy separation) and high anastomotic tension. Conversely, achieving closure at common commissure demands superior surgical expertise.

In response to this, several modified surgical techniques, esophageal rotation and right closure combined with a common opening (7), the Later-cut-overlap method (8), and the self-pulling and latter transection (SPLT) method (9), have been developed. While these techniques have optimized the operation to some extent, complications remain unavoidable. There are limited direct reports on managing “pseudo-stoma” after its appearance, with most cases requiring reopening and reanastomosis, further shortening the distal esophagus. This increases the difficulty of laparoscopic operation and necessitates an open chest or thoracoscopic operation for completing the anastomosis again. A report (10) suggests that assisted percutaneous transdiaphragmatic approach plus laparoscopic gastrectomy can reduce intraoperative anastomotic complications. AEG may employ laparoscopic and thoracoscopic procedures as effective modalities, but further clinical validation is necessary. In this instance, the patient experienced a pseudo-stoma, with the anastomosis situated approximately 36cm from the incisor. If extraction of the distal esophagus and lower anastomosis were to be performed via thoracotomy, it would necessitate trimming of the small intestine mesentery to increase its length and reduce tension, facilitating easier mobilization and anastomosis of the esophageal stump (11). This procedure is intricate and entails significant trauma. After a multidisciplinary team (MDT) consultation, which included endoscopic, thoracic, and gastrointestinal surgeons, it was determined that the size of the esophagojejunal anastomosis created by the intraoperative laparoscopic linear stapler closure was appropriate. It was also concluded that the patency of the anastomosis could be restored by mucosal incision of the food pipe stump using an endoscope. However, it is important to note that the endoscopist may inadvertently transect the stapled segment of the esophageal stump (mistakenly perceiving it as the obstructed segment) (**Figure 3A**). Hence, it is essential to undergo an incision procedure under the supervision of a gastrointestinal surgeon. Moreover, in light of the potential risk of postoperative anastomotic leak/stenosis and the need to ensure prompt enteral nutrition, surgical endoluminal magnets (SEMs) and feeding tubes were also inserted during the procedure. The patient was discharged from hospital after smooth recovery and follow-up endoscopy showed slight narrowing at anastomosis without subjective sensation obstruction after eating.

## Conclusion

In laparoscopic total gastrectomy, the atresia anastomosis of “pseudo” lumen between jejune and esophagus occurs commonly and has significant complication. In this instance, effective resolution was achieved by performing an endoscopic incision on the esophageal mucosa at the site of the “pseudo” lumen’s anastomosis, along with inserting self-expanding covered metal stents (SEMs), thus eliminating the necessity for thoracic reconstruction. Several points merit attention regarding this case: Firstly, whether increasing both the length and width of the incision in the “pseudo” lumen mucosal (**Figure 3B**) could potentially lower the risk of anastomotic stenosis; secondly, further investigation is required to determine the ideal timing for stent removal. This case serves as a warning to surgeons about meticulously managing details during esophago-jejunal anastomosis to prevent intraoperative complications. Additionally, it offers a novel approach for straightforward, safe, and effective intervention when faced with atresia and anastomosis issues within this context during surgery.

## Data Availability

The original contributions presented in the study are included in the article/supplementary material, further inquiries can be directed to the corresponding author/s.

## References

[1] Manabe N Matsueda K Haruma K. Epidemiological review of gastroesophageal junction adenocarcinoma in Asian countries. Digestion. (2022) 103:29–36. doi: 10.1159/000519602 34718236

[2] Hasegawa S Yoshikawa T. Adenocarcinoma of the esophagogastric junction: incidence, characteristics, and treatment strategies. Gastric Cancer. (2010) 13:63–73. doi: 10.1007/s10120-010-0555-2 20602191

[3] Huang CM Lv CB Lin JX Chen QY Zheng CH Li P. Laparoscopic-assisted versus open total gastrectomy for Siewert type II and III esophagogastric junction carcinoma: a propensity score-matched case-control study. Surg Endosc. (2017) 31:3495–503. doi: 10.1007/s00464-016-5375-y 27981384

[4] Kurokawa Y Sasako M Sano T Yoshikawa T Nashimoto A. Ten-year follow-up results of a randomized clinical trial comparing left thoracoabdominal and abdominal transhiatal approaches to total gastrectomy for adenocarcinoma of the oesophagogastric junction or gastric cardia. Br J Surg. (2015) 102:341–8. doi: 10.1002/bjs.9764 PMC5024022 25605628

[5] Kurokawa Y Takeuchi H Doki Y Mine S Terashima M Yasuda T. Mapping of lymph node metastasis from esophagogastric junction tumors: A prospective nationwide multicenter study. Ann Surg. (2021) 274:120–7. doi: 10.1097/SLA.0000000000003499 31404008

[6] Inaba K Satoh S Ishida Y Taniguchi K Isogaki J Kanaya S. Overlap method: novel intracorporeal esophagojejunostomy after laparoscopic total gastrectomy. J Am Coll Surg. (2010) 211:e25–9. doi: 10.1016/j.jamcollsurg.2010.09.005 21036074

[7] Wei MG Zhou S Zhang B Yang Y Wang K Gao P. Overlap esophagojejunostomy with multi-mode modifications in totally laparoscopic total gastrectomy: safety and feasibility of 152 cases from a single center. Zhonghua Wei Chang Wai Ke Za Zhi. (2022) 25:433–9. doi: 10.3760/cma.j.cn441530-20220309-00098 35599398

[8] Huang CM Huang ZN Zheng CH Li P Xie JW Wang JB. An isoperistaltic jejunum-later-cut overlap method for esophagojejunostomy anastomosis after totally laparoscopic total gastrectomy: A safe and feasible technique. Ann Surg Oncol. (2017) 24:1019–20. doi: 10.1245/s10434-016-5658-5 27921193

[9] Hong J Wang YP Wang J Bei YB Hua LC Hao HK. A novel method of self-pulling and latter transected reconstruction in totally laparoscopic total gastrectomy: feasibility and short-term safety. Surg Endosc. (2017) 31:2968–76. doi: 10.1007/s00464-016-5314-y 27826782

[10] Huang Y Liu G Wang X Zhang Y Zou G Zhao Z. Safety and feasibility of total laparoscopic radical resection of Siewert type II gastroesophageal junction adenocarcinoma through the left diaphragm and left thoracic auxiliary hole. World J Surg Oncol. (2021) 19:73. doi: 10.1186/s12957-021-02183-9 33714262 PMC7956135

[11] Mariette C Piessen G Briez N Gronnier C Triboulet J. Oesophagogastric junction adenocarcinoma: which therapeutic approach? Lancet Oncol. (2011) 12:296–305. doi: 10.1016/S1470-2045(10)70125-X 21109491

